# Regulation, overexpression, and target gene identification of *Potato Homeobox 15* (*POTH15*) – a class-I *KNOX* gene in potato

**DOI:** 10.1093/jxb/erw205

**Published:** 2016-05-23

**Authors:** Ameya S. Mahajan, Kirtikumar R. Kondhare, Mohit P. Rajabhoj, Amit Kumar, Tejashree Ghate, Nevedha Ravindran, Farhat Habib, Sundaresha Siddappa, Anjan K. Banerjee

**Affiliations:** ^1^Biology Division, Dr. Homi Bhabha Road, IISER Pune, Pune - 411008, Maharashtra, India; ^2^School of Biology, IISER TVM, Thiruvananthapuram (Trivandrum) - 695016, Kerala, India; ^3^Dept. of Botany, SPP University (formerly University of Pune), Pune - 411007, Maharashtra, India; ^4^Biological Sciences, IISER Bhopal, Bhopal - 462066, Madhya Pradesh, India; ^5^Division of Crop Improvement, Central Potato Research Institute, Shimla - 171001, India

**Keywords:** KNOX target genes, leaf development, photoperiod, *Potato Homeobox 15* (*POTH15*), RNA sequencing, shoot apical meristem, *Solanum tuberosum*.

## Abstract

*Potato Homeobox 15* (*POTH15*) is a *KNOX-I* (*Knotted1*-like homeobox) family gene in potato that is orthologous to *Shoot Meristemless* (*STM*) in Arabidopsis. Despite numerous reports on *KNOX* genes from different species, studies in potato are limited. Here, we describe photoperiodic regulation of *POTH15*, its overexpression phenotype, and identification of its potential targets in potato (*Solanum tuberosum* ssp. *andigena*). qRT-PCR analysis showed a higher abundance of *POTH15* mRNA in shoot tips and stolons under tuber-inducing short-day conditions. *POTH15* promoter activity was detected in apical and axillary meristems, stolon tips, tuber eyes, and meristems of tuber sprouts, indicating its role in meristem maintenance and leaf development. *POTH15* overexpression altered multiple morphological traits including leaf and stem development, leaflet number, and number of nodes and branches. In particular, the rachis of the leaf was completely reduced and leaves appeared as a bouquet of leaflets. Comparative transcriptomic analysis of *35S::GUS* and two *POTH15* overexpression lines identified more than 6000 differentially expressed genes, including 2014 common genes between the two overexpression lines. Functional analysis of these genes revealed their involvement in responses to hormones, biotic/abiotic stresses, transcription regulation, and signal transduction. qRT-PCR of selected candidate target genes validated their differential expression in both overexpression lines. Out of 200 randomly chosen POTH15 targets, 173 were found to have at least one tandem TGAC core motif, characteristic of KNOX interaction, within 3.0kb in the upstream sequence of the transcription start site. Overall, this study provides insights to the role of *POTH15* in controlling diverse developmental processes in potato.

## Highlight

This study demonstrates that overexpression of *POTH15* (a class-I *KNOX* in potato) can alter multiple morphological traits, and identifies numerous POTH15 targets involved in diverse developmental processes in potato.

## Introduction

*Knotted1*-like homeobox (*KNOX*) genes are ubiquitous in green plants and are involved in cell fate determination and development. The first *KNOX* gene to be discovered was *Knotted1* (*Kn1*) from maize, and it was shown to regulate the maintenance of the shoot apical meristem (SAM ) (Vollbrecht *et al.*, 1991). Since then a number of studies have identified *KNOX* genes from diverse plant species. In higher plants, *KNOX* genes form a multimember family of transcription factors. Based on the *KNOX* expression pattern and intron positions, they are grouped into two sub-classes; class I and class II (Kerstetter *et al.*, 1994). A number of *KNOX* overexpression and null mutant studies have shown that class-I *KNOX* genes regulate various vegetative and reproductive developmental processes, such as SAM maintenance, leaf development, floral development, tuber formation, and bulbil formation (Rosin *et al.*, 2003; Abraham-Juarez *et al.*, 2010; Hay and Tsiantis, 2010). In plants with simple leaves such as Arabidopsis, *KNOX* genes are expressed only in the meristem and stem, whereas in compound leaf species such as *Cardamine hirsuta* and tomato, they are expressed in leaf primordia as well (Hareven *et al.*, 1996; Bharathan *et al.*, 2002; Barkoulas *et al.*, 2008; Piazza *et al.*, 2010). Ectopic expression of *KNOX-I* in leaf primordia results in various phenotypes ranging from leaf serration to compounding depending on the extent of expression (Hake *et al.*, 2004).

Ectopic expression of *KNOX* genes has been shown to cause severe pleotropic effects and thus exclusion of their expression outside their regular domain is critical for normal plant development (Giacomo *et al.*, 2013). Several genes are required for repression of *KNOX* genes in leaves. Among them, the MYB (myeloblastosis) transcription factor ROUGH SHEATH2 (RS2) in maize, its Arabidopsis putative ortholog ASYMMETRIC LEAVES1 (AS1), and the plant-specific lateral organ boundaries (LOB) family protein ASYMMETRIC LEAVES2 (AS2) have been well studied. A loss-of-function mutation in these genes results in ectopic expression of *KNOX* genes in leaves (Timmermans *et al.*, 1999; Tsiantis *et al.*, 1999; Ori *et al.*, 2000; Semiarti *et al.*, 2001; Byrne *et al.*, 2002; Iwakawa *et al.*, 2002). These genes are suggested to establish and/or maintain the repressed state of *KNOX* loci via chromatin remodelling. Additionally, activities of auxin, YABBY, and polycomb repressor complex (PRC) genes are shown to repress *KNOX* gene expression in leaves (Kumaran *et al.*, 2002; Katz *et al.*, 2004; Hay *et al.*, 2006).

Another group of transcription factors, BEL1-like homeodomain (BELL) proteins, is known to interact with KNOX protein partners to regulate the expression of numerous target genes in potato (Chen *et al.*, 2003, 2004; Sharma *et al.*, 2014). Recently, Sharma *et al.* (2016) have identified a large number of BEL5 target genes in potato. Since *KNOX* genes act as transcription factors (TFs), identification of their targets genes would be crucial to understand their function. Only a handful of studies have reported the genes targeted by *KNOX* TFs. Several studies have demonstrated that *KNOX* genes directly target *GA20ox1* [gibberellin (GA) biosynthesis gene] and down-regulate its activity, resulting in reduction of GA levels (Sakamoto *et al.*, 2001; Hay *et al.*, 2002; Chen *et al.*, 2003, 2004; Kessler *et al.*, 2006). *KNOX* up-regulates the expression of *GA2ox1* (a GA catabolic gene), resulting in low GA levels in the tissue (Hay *et al.*, 2003; Bolduc and Hake, 2009). *KNOX* also regulates cytokinin and auxin levels and biosynthesis of lignin (Hewelt *et al.*, 2000; Frugis *et al.*, 2001; Hertzberg *et al.*, 2001; Hay *et al.*, 2003, 2006; Mele *et al.*, 2003; Yanai *et al.*, 2005; Du *et al.*, 2009; Bolduc *et al.*, 2012). Bolduc *et al.* (2012) have identified several direct targets of *Kn1* (a class-I *KNOX*) including other homeobox genes and numerous hormone metabolism genes in maize. Recently, Tsuda *et al.* (2014) showed that a rice *KNOX-I* gene *OSH1* represses the brassinosteroid (BR) phytohormone pathway through activation of BR catabolism genes, and they concluded that local control of BR levels by *KNOX* genes could be a key regulatory step in SAM function. In summary, these studies establish the importance of *KNOX* in plant development and reproduction.

Our focus here is to understand the role of *KNOX* genes in potato development. Previously, *POTH1* (a class-I *KNOX* gene) was shown to regulate vegetative development and tuberization in potato, and its mRNA was found to be phloem-mobile (Rosin *et al.*, 2003; Mahajan *et al.*, 2012). In preliminary work, we identified full-length transcript sequences of six *KNOX* genes in potato (*Solanum tuberosum* ssp. *andigena* 7540). Of them, *POTATO HOMEOBOX 15* (*POTH15*; a class-I *KNOX* and an ortholog of *STM* in Arabidopsis) is the focus of this study. We have examined the photoperiodic regulation of *POTH15* and the effect of its overexpression, and we have identified potential *POTH15* target genes in potato. Our results indicate that *POTH15* mRNA abundance is affected by photoperiod and its promoter has a widespread expression pattern. Overexpression of *POTH15* drastically alters plant morphology and a comparative RNA-sequencing analysis revealed more than 6000 putative target genes of POTH15, suggesting its role in diverse developmental processes in potato. In addition, from a random screen of 200 targets, ~87% of them had at least one tandem TGAC core motif in the upstream sequence within 3.0kb of the transcription start site, suggesting a possible KNOX interaction with their target genes.

## Materials and methods

### Plant material and growth conditions

Potato (*Solanum tuberosum* ssp. *andigena* 7540 and *S. tuberosum* cv. Désirée) and tobacco (*Nicotiana tabacum* cv. Petite Havana) were used as model systems in this study. *In vitro* cultures of all the plants were maintained at 22±1 °C with light intensity of 300 mmol m^−2^ s^−1^ in a growth incubator (Percival Scientific, USA) with either a long day (LD; 16 h light, 8 h dark) or short day (SD; 8 h light, 16 h dark) photoperiod, depending on the experimental treatment. Soil-grown plants of potato and tobacco were maintained at 300 μmol m^−2^ s^−1^ light intensity with 22±1 °C day temperature and 20 °C night temperature in a growth chamber (Percival Scientific, USA) under either a LD or SD photoperiod, depending on the experimental treatment.

### Identification and validation of *POTH15*

A putative sequence for *POTH15* mRNA was derived from the Potato Genome Sequence Consortium (PGSC) database (http://solanaceae.plantbiology.msu.edu/cgi-bin/annotation_report.cgi) and the expression of *POTH15* was validated by reverse transcription PCR (RT-PCR). Total RNA from leaves of 8-week-old potato plants was extracted with Trizol (Invitrogen) and RT-PCR was performed using a one-step RT-PCR kit (Invitrogen) with the primer pair POTH15-RTF and POTH15-RTR (Supplementary Table S1 at *JXB* online). Reaction conditions were 55 °C for 60min, 95 °C for 2min, followed by 30 cycles of 95 °C for 30s, 55 °C for 30s, and 72 °C for 2min. As the potato genome was not available when the present investigation commenced, 5′ rapid amplification of cDNA ends (RACE) was performed to obtain the full-length transcript sequence of *POTH15* using a Clontech SMARTer RACE kit (cat. no. 634923) following the manufacturer’s instructions. The primers used for RACE were c-P15-5′RACE and n-P15-5′RACE (Supplementary Table S1). Based on the sequence obtained from RACE, the full-length *POTH15* gene was amplified from leaf total RNA using the primer pair POTH15-FLF and POTH15-FLR, and cloned into the sub-cloning vector pGEMTeasy (Promega). Similarly, five more novel *KNOX* genes were amplified from potato (*S. tuberosum* ssp. *andigena* 7540) and their transcript sequences were submitted to NCBI (Supplementary Fig. S1). A phylogenetic tree for all these potato *KNOX* genes was generated by the neighbor-joining method using MEGA6 (Tamura *et al.*, 2013).

### Construct design and plant transformations

The full-length *POTH15* gene sequence was amplified using the primers P15FL-FP-XbaI and P15FL-RP-KpnI and cloned downstream of *CaMV35S* promoter in the binary vector pCAMBIA1300 to generate a *35S*::*POTH15* construct. The DNA sequence upstream of *POTH15* CDS (coding DNA sequence) was obtained from the PGSC database (http://solanaceae.plantbiology.msu.edu/cgi-bin/annotation_report.cgi). The *POTH15* promoter (1620bp) including 5′ UTR (296bp) was amplified from potato genomic DNA using the primers Pr15RE187F2 and pr15RE-FLR2 (Supplementary Table S1) and was cloned into binary vector pBI121 to generate a *promPOTH15::GUS* construct. The constructs (*35S::POTH15-pCAMBIA1300*, *35S::GUS-pBI101*, and *promPOTH15::GUS-pBI121*) were transformed to *Agrobacterium tumefaciens* strain GV2260. *Agrobacterium*-mediated transformation of *S. tuberosum* ssp. *andigena* and cv. Désirée was carried out by the method described in Banerjee *et al.* (2006), and in tobacco by the method of Horsch *et al.* (1985).

### Expression analysis of *POTH15* by qRT-PCR

For tissue-specific expression analysis of *POTH15*, potato (*S. tuberosum* ssp. *andigena* 7540) plants were used. Sixteen plants were transferred to soil and maintained under LD photoperiod for 8 weeks. Half of the plants were then transferred to tuber-inducing SD conditions, whilst the remaining plants were maintained under LD conditions. Different tissues (leaf, petiole, shoot tip, stem, and stolon) were harvested 15 d post SD/LD induction. Tissues were frozen in liquid nitrogen immediately after harvest and stored at –80 °C until further use. Total RNA was isolated from the frozen tissue using the TRizol (Invitrogen) method as per the manufacturer’s instructions. One microgram of total RNA was reverse-transcribed using MMLV-RT (Promega) and gene-specific primers POTH15-qR2 for POTH15 and 18S-rRNA-RP for 18S-rRNA. qPCRs were performed on a Mastercylcer ep Realplex using the primers POTH15-qF2 and POTH15-qR2 for *POTH15*, and 18S-rRNA-FP2 and 18S-rRNA-RP for *18S rRNA* (Supplementary Table S1). The reactions were carried out using a KAPA SYBR green master mix (Kapa Biosystems) and incubated at 95 °C for 2min followed by 40 cycles of 95 °C for 15s and 60 °C for 30s. 18S-rRNA was used for normalization for all the reactions. PCR specificity was checked by melting curve analysis, and data were analyzed using the 2^–ΔΔCt^ method (Livak and Schmittgen, 2001).

### *POTH15* transcript abundance in overexpressing lines

Four *POTH15* overexpressing (OE) lines (G8, G9, E2-13, and E2-13), wild-type, and *35S::GUS* (control) lines were grown in soil for 12 weeks under LD conditions in a plant growth chamber (Percival Scientific, Ltd). Out of 12 independent plants for each line, shoot apexes (3–4cm) from six plants were pooled for harvest (i.e. forming two biological replicates). Total RNA was isolated using TRizol (Invitrogen). Two microgram of RNA was reverse-transcribed using the oligo(dT) primer and SuperScript-III reverse transcriptase (Invitrogen). qPCRs were performed on a Mastercylcer ep Realplex using gene-specific primers (Supplementary Table S1). Relative mRNA levels of *POTH15* in all OE lines were measured with respect to wild-type and *35S::GUS* plants. qRT-PCR data were analyzed by Student’s *t*-test (at *P*<0.05) using GraphPad Prism (6.07 version).

### Morphometric analysis

Wild-type and *35S*::*POTH15* lines of potato (*andigena*) were transferred to soil and maintained in a growth chamber for 10 weeks under LD conditions. Plant height, number of nodes and leaves per plant, internodal distance, and leaflet number per leaf were measured for all the plants. The data were plotted using the software Graph – Version 4.4.2 (www.padowan.dk).

### Tuberization assay

To investigate the tuber yield, two *POTH15* OE lines (G8 and E2-13) and wild-type plants (12 plants for each line) were grown in soil under LD conditions for 8 weeks in a plant growth chamber (Percival Scientific, Ltd). Half of the plants from each line were then subjected for 4 weeks to either LD or SD induction. The tuber yields, measured as gram fresh weight per plant, were recorded at the end of induction. The tuber yields data were analyzed by one-way ANOVA. Error bars represent (±) standard deviation for six biological replicates.

### Histology

For histology, leaves and stems of both wild-type and *35S::POTH15* lines that were grown for 10 weeks in soil under LD conditions were fixed using 4% paraformaldehyde in phosphate-buffered saline (PBS) (0.1M, pH 7). The blocks of fixed tissues were prepared in 4% agarose in PBS (w/v) and then sectioned on a vibratome (Leica).

### Analysis of GUS activity

GUS assay (Jefferson, 1987) was done by incubating the tissue samples of *promPOTH15::GUS* potato lines in assay buffer containing 1M NaPO_4_, pH 7; 0.25M EDTA, pH 8; 10% TritonX 100; 1mM 5-bromo-4-chloro-3-indolyl-β-D-GlcUA, 0.5mM potassium ferricyanide and 0.5mM potassium ferrocyanide. Samples were cleared with 100% ethanol and photographed under a Leica stereo microscope S8AP0 or a Zeiss compound microscope.

### RNA isolation, library preparation and RNA sequencing

For the RNA-sequencing experiment, *POTH15* OE lines (G8 and E2-18) and *35S::GUS* lines (control) were grown in soil for 12 weeks under LD conditions in a plant growth chamber (Percival Scientific, Ltd). To avoid bias as well as to reconfirm the RNA sequencing data, we selected two different transgenic lines having high (E2-13) and moderate (G8) levels of *POTH15* expression (see results) for our RNA sequencing study. Shoot apexes (4–5cm) from 18 independent plants were harvested and samples were pooled from six plants to form three biological replicates per line. The total RNA was isolated using Trizol (Invitrogen). The RNA samples were quantified on Qubit using Qubit RNA HS kit (Invitrogen). Ten micrograms of RNA were taken and the poly (A) RNA was enriched using a Dynabeads® mRNA direct microkit (Ambion) following the manufacturer’s instructions. The libraries were prepared using an Ion Total RNA-Seq V2 kit (Life Technologies). The quality and concentrations of the libraries was determined using a DNA1000 chip on a Bioanalyzer (Agilent). Libraries were then sequenced on the Ion Proton^TM^ platform. The reads were obtained in FastQ format and quality was checked using FastQC (http://www.bioinformatics.bbsrc.ac.uk/projects/fastqc/). RNA-sequence data analyses were performed using the Tuxedo suite. The reads were aligned to a potato reference genome sequence (PGSC_DM_v3.4_gene.fasta.zip, at http://solanaceae.plantbiology.msu.edu/pgsc_download.shtml) using Bowtie 2.0 (Langmead *et al.*, 2009) and TopHat-Version 2.0.13 (Kim *et al.*, 2011) software with default parameters. The reads that aligned to the genome were quantified by the Cuffquant and Cufflinks programs (Trapnell *et al.*, 2013), which provided relative abundance values by calculating fragments per kilobase of exon per million fragments mapped (FPKM) (Mortazavi *et al.*, 2008, Mizrachi *et al.*, 2010). Cufflinks was also used to find isoforms, promoters, translation start sites, and sites of alternative splicing. The differential expression analysis of genes was performed using Cuffdiff package-2.2.1 (Trapnell *et al.*, 2013). The Cuffdiff results were compiled and visualized using the R package CummeRbund,Version 2.0 (http://bioconductor.org/packages/release/bioc/html/cummeRbund.html). Gene ontology (GO) analyses were performed using the Blast2GO software v1.3.3 for the functional annotation of differentially expressed genes (Conesa *et al.*, 2005; Götz *et al.*, 2008). The FASTA file containing the transcript sequences of all unique differentially expressed (DE) genes were cloud-blasted using the BlastX program against non-redundant protein database (NCBI) in the Blast2GO software (parameters for cloud-blast: sequence length ≥100bp; number of blast hits, 20; e-value, 10; HSP length cut-off, 33). The mapping tool was used to obtain GO information from retrieved database matches. GO term-mapping was done with a sequence length ≥100bp. Annotation of all sequences was performed using the annotation tool against filter GO by taxonomy to green plants, with the following parameters: sequence length ≥150bp; e-value Hit Filter set to 3; annotation cut-off set to 25; GO weight set constantly to 5. GO term-based classification charts were also generated using the Blast2GO software.

### Validation of POTH15 targets by qRT-PCR

Total RNA was isolated from the shoot apex samples harvested for the RNA-sequencing experiment (35S::GUS and two *POTH15* OE lines, G8 and E2-13), with three biological replicates. As RNA sequencing was performed on shoot apex samples of two *POTH15* OE lines and 35S::GUS plants, the same lines (aliquot samples) were used for POTH15 target validation analyses. These plants were grown in soil for 12 weeks under LD conditions in a plant growth chamber (Percival Scientific, Ltd). Four micrograms of the total RNA were used for cDNA synthesis using the oligo (dT) primer and SuperScript-III reverse transcriptase (Invitrogen). qPCRs were performed on a Mastercylcer ep Realplex using gene-specific primers (Supplementary Table S1). The reactions were carried out using the KAPA SYBR green master mix (Kapa Biosystems) and incubated at 95 °C for 2min followed by 40 cycles of 95 °C for 15s and 60 °C for 30s. *StActin* was used for normalization for all the reactions. PCR specificity was checked by melting curve analysis, and data were analyzed using the 2^–ΔΔCt^ method (Livak and Schmittgen, 2001). Data from POTH15 target validation experiments were analyzed by Student’s *t*-test (at *P*<0.05) using GraphPad Prism (6.07 version). The list of primer sets used for the qRT-PCR analysis of POTH15 targets is provided in Supplementary Table S1.

### Identification of tandem TGAC core motifs in the promoter sequences of POTH15 targets

To identify the TGAC core motif (a characteristic of the KNOX/BEL interaction; Chen *et al.*, 2004) in the upstream sequences of POTH15 targets, 200 genes were randomly selected from the differentially expressed (DE) common genes between two *POTH15* OE lines (G8 and E2-13). The promoter sequences within 3.0kb of the transcription start site (TSS) for the 200 genes were manually retrieved from PGSC database (http://solanaceae.plantbiology.msu.edu/cgi-bin/gbrowse/potato). The presence of tandem TGAC core motifs in the promoter sequences of targets was identified using the RSAT tool (van Helden, 2003; Thomas-Chollier *et al.*, 2008, 2011; Medina-Rivera *et al.*, 2015) as per the four possible combinations described in Sharma *et al.* (2016), and a maximum linker length between two motifs of 30 nucleotides was selected. Similarly, for controls, the promoter sequences for 15 random non-DE genes were also searched for the presence of tandem TGAC core motifs. For the 200 POTH15 targets, correlation analysis (at *P*<0.05) using GraphPad Prism (6.07 version) for fold change versus number of tandem TGAC core motifs was also performed. Additionally, a statistical analysis for the presence of average numbers of tandem TGAC core motifs between DE and non-DE genes was also performed. Data were analyzed by Student’s *t*-test using GraphPad prism 7 (*n*=200; *P*<0.05). In the results, **** represents a significant difference at *P*≤0.001.

## Results

### *POTH15* belongs to a *KNOX-I* family

**Fig. 1.**
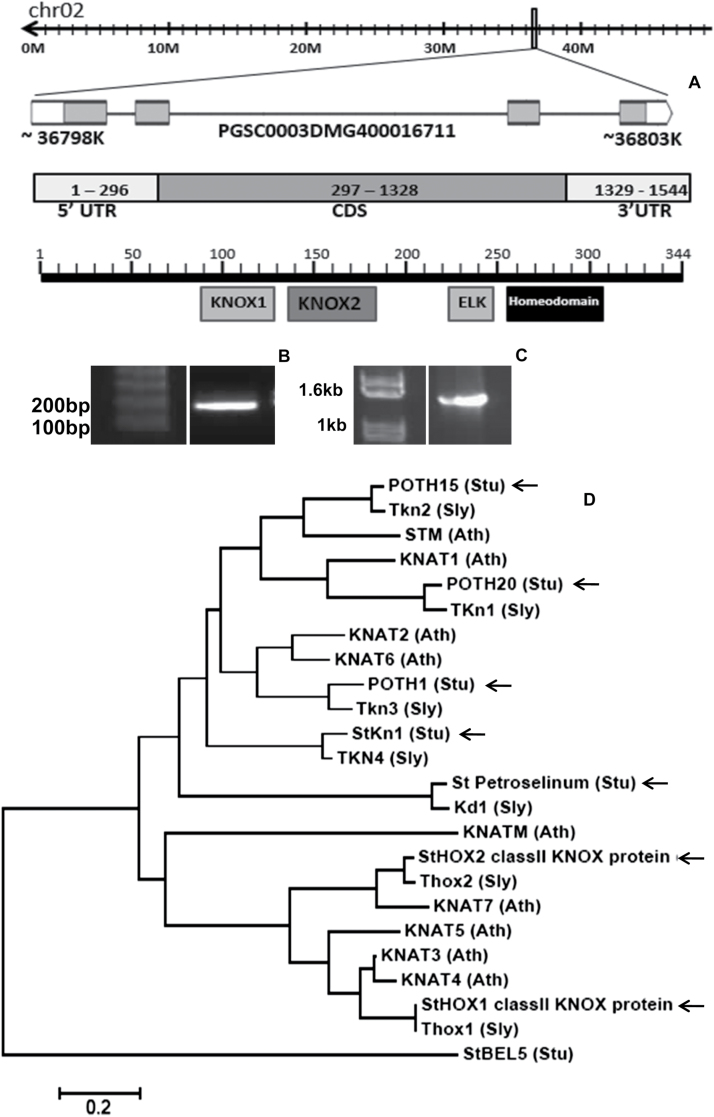
(A) Identification and validation of *POTH15*. Location of the *POTH15* gene in the potato genome, with structure of the *POTH15* transcript and domains in the POTH15 protein. (B) RT-PCR to validate the presence of the *POTH15* transcript, and (C) amplification of full-length *POTH15* mRNA from potato shoot tips. (D) Phylogenetic tree for KNOX proteins from potato, tomato, tobacco, and Arabidopsis, generated by the neighbor-joining method using MEGA6 (Tamura *et al.*, 2013). The tree is rooted to the *St*BEL5 sequence. The branches indicate the number of amino acid substitution per site (see scale bar). Arrows indicate KNOX members in potato.

Based on the homology search for *KNOX* genes in the potato genome, the *STM* ortholog in potato was identified and labelled as *POTH15* (Fig. 1A; NCBI accession no. KJ477687). RT-PCR analysis followed by sequence verification validated the presence of *POTH15* mRNA in potato, and the sequence confirmed it to be a *KNOX-I* gene (Fig. 1B). 5′ RACE identified the full-length *POTH15* mRNA sequence (Fig. 1C). Further analysis within the PGSC database revealed that *POTH15* is located on chromosome number two of potato, it has a transcript length of 1544 bases, and it is predicted to code for a 343 amino acid protein. Additionally, our work identified five more *KNOX* genes from the potato genome (Supplementary Fig. S1), thereby bringing the total number of *KNOX* genes identified in potato to seven. A phylogenetic tree of these seven potato *KNOX* proteins shows that four of them belong to class I, two to class II, and one is a mini-*KNOX* (Fig. 1D).

### Transcript abundance of *POTH15* is photoperiod regulated

**Fig. 2.**
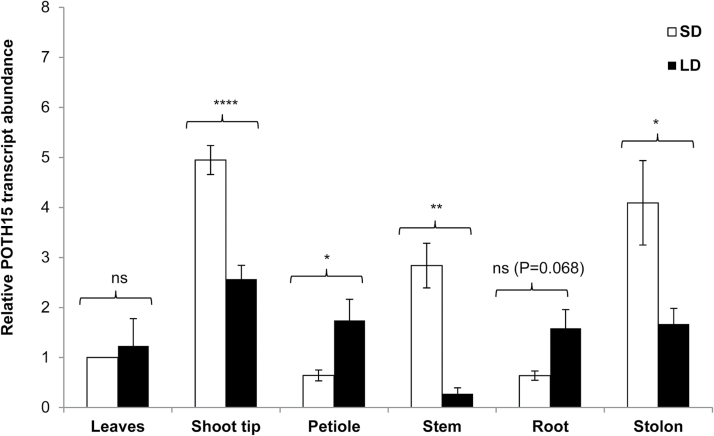
Tissue-specific abundance of POTH15. *POTH15* transcript abundance in leaves, shoot tips, petioles, stems, roots and stolons of wild-type *S. tuberosum* ssp*. andigena* plants grown under SD and LD conditions for 15 d. Sixteen plants were transferred to soil and maintained under LD photoperiod for 8 weeks. Different tissues were harvested 15 d post SD/LD induction. Total RNA (1 µg) was reverse-transcribed using MMLV-RT and the gene-specific primers POTH15-qR2 for *POTH15* and 18S-rRNA-RP for *18S-rRNA*. qPCRs were performed on a Mastercylcer ep Realplex using primers POTH15-qF2 and POTH15-qR2 for *POTH15*, and 18S-rRNA-FP2 and 18S-rRNA-RP for *18S rRNA*. 18S-rRNA was used for normalization for all the reactions. Data were analyzed using the 2^–ΔΔCt^ method (Livak and Schmittgen, 2001). Data are mean ± standard deviations for three biological replicates. Fold-change in *POTH15* transcript levels in different tissue types were calculated with respect to its level in leaves under SD conditions. * Represents significant difference at *P*≤0.05, ** *P*≤0.01, **** at *P*≤0.0001. ns = not significant at *P*<0.05.

To investigate if photoperiod has any effect on abundance of the *POTH15* transcript, we examined the relative levels of *POTH15* mRNA in photoperiod-responsive wild-type potato (*andigena*) plants grown under both SD and LD conditions. *POTH15* mRNA levels were quantified in shoot tips, leaves, petioles, stems, roots, and stolons through qRT-PCR analysis (Fig. 2). Among the tissue types evaluated, shoot tips, stolons, and stems showed higher abundance of *POTH15* mRNA under tuber-inducing SD conditions, whereas petiole and roots exhibited higher accumulation of *POTH15* mRNA under LD conditions compared to SD conditions. However, leaves did not show any significant changes in mRNA abundance under either SD or LD photoperiods (Fig. 2).

### Promoter activity of *POTH15*

**Fig. 3.**
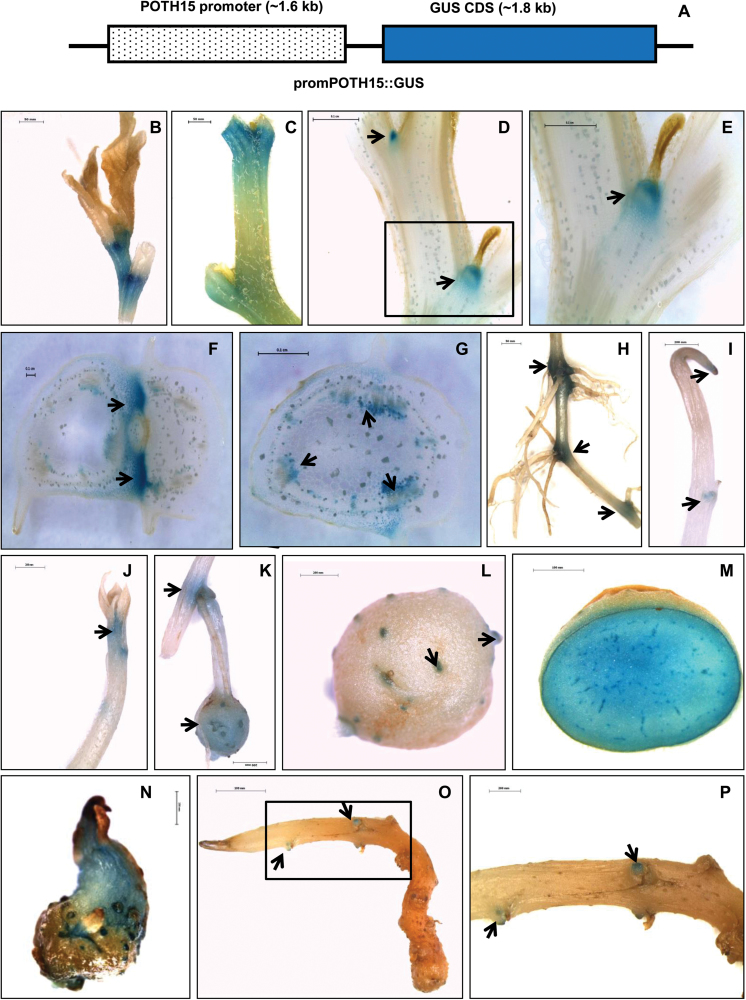
The *POTH15* promoter expression. The _*prom*_ *POTH15::GUS* construct (A). Promoter activity of *POTH15* in the shoot apex (B), nodes (C), at lateral root initiation (H, arrows), stolon tip (I), stolon meristem (J), root–stolon junction (K, arrows), mini-tuber (K, arrow), tuber eyes (L, arrows), tuber pith (M), meristems of 2-week-old (N) and 4-week-old excised tuber sprouts of *S. tuberosum* cv. Désirée (O, P; where P is a magnified image of O, highlighted region). L.S. of nodal and internodal region showing *POTH15* promoter activity in axillary meristem (D, E; where E is a magnified image of D, highlighted region). T.S. of nodal region (F) and internodal region showing *POTH15* promoter activity in vascular tissues (G). Scale bars: (C, H) 50mm; (F, G, M, P) 100mm; (B, D, E, I–L, N, O) 200mm. Arrows indicate the regions of *POTH15* promoter activity.

A *GUS* assay for _*prom*_ *POTH15::GUS* transgenic lines of potato (cv. Désirée) detected *POTH15* promoter activity in the shoot apex (Fig. 3B), axillary nodes (Fig. 3C), at lateral root initiation (Fig. 3H), stolon tips (Fig. 3I), stolon meristem (Fig. 3J), root–stolon junction and mini-tuber (Fig. 3K), tuber eyes (Fig. 3L), tuber pith (Fig. 3M), and in the meristems of 2- and 4-week-old excised tuber sprouts (Fig. 3N–P). Moreover, longitudinal and transverse sections comprising nodal and internodal regions of the stem showed promoter activity in axillary meristems and in vascular tissues, respectively (Fig. 3F, G). The promoter sequence was analyzed for the presence of *cis*-regulatory elements using the promoter analysis software plantPAN (Chang *et al.*, 2008) and PLACE (Higo *et al.*, 1999). Several light regulatory elements such as GATA, GT-1, and I-boxes were detected in the *POTH15* promoter. The plantPAN software also identified the presence of binding sites for several other transcription factors, such as MYB, AthB1, AthB5, AtHB9, AGL15, and AGL3 (Supplementary Table S2), in the *POTH15* promoter.

### Overexpression of *POTH15* alters multiple morphological traits

**Fig. 4.**
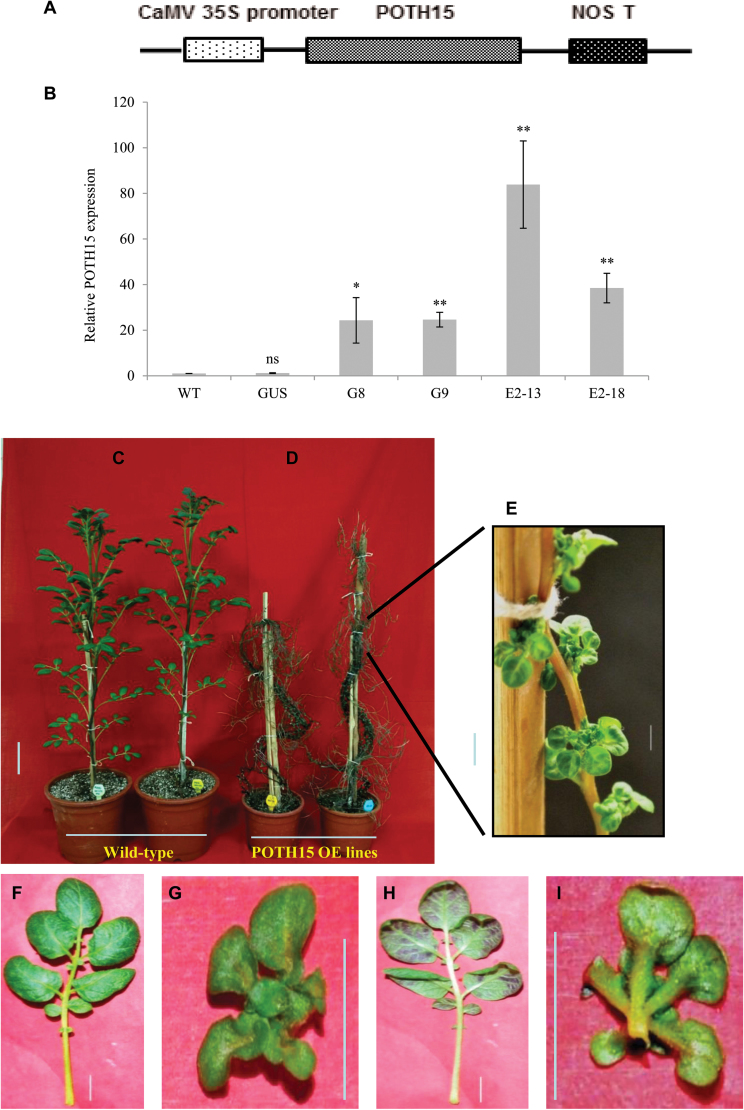
*POTH15* overexpression (OE) drastically changes the plant architecture. The *35S::POTH15* construct (A). Relative mRNA levels of *POTH15* in OE lines (G8, G9, E2-13, and E2-18) is shown with respect to controls, wild-type (WT), and *35S::GUS* (GUS), from 12-week-old soil-grown plants under LD conditions (B). *StActin* mRNA was used as reference for normalization of qRT-PCR. The fold-change in RNA levels was calculated as the 2^–ΔΔCt^ value relative to the mean values in WT sample. Data represent means ± standard deviations for two biological replicates, where shoot apexes from six independent plants were pooled for one biological replicate from each line. Data were analyzed by *t*-test separately for WT and each transgenic line. * and ** represent significant difference at *P*≤0.05 and *P*≤0.01, respectively. ns = not significant. Twelve-week-old wild-type potato plants (C) and *POTH15* OE lines (D, E; where E is a close-up of D). The dorsal and ventral view of the leaves from wild-type (F, H) and *POTH15* OE (G, I) lines. Scale bars: (C, D) 5cm; (E–I) 1cm.

In order to investigate the role of *POTH15* in development, *35S::POTH15* transgenic lines were generated (the construct design is shown in Fig. 4A) and five independent lines (G8, G9, G11, E2-13, and E2-18) were selected for further analysis. *POTH15* OE lines and controls (wild-type and *35S::GUS*) were grown for 12 weeks and relative *POTH15* mRNA levels in all these lines were measured by qRT-PCR. As shown in Fig. 4B, OE line E2-13 showed the highest transcript accumulation, followed by line E2-18. The lines G8 and G9 showed moderate levels of *POTH15* transcript accumulation (Fig. 4B). Overexpression lines exhibited severe morphological changes in plant architecture. The *35S::POTH15* lines were slender compared to wild-type plants (Fig. 4C, D) and these lines exhibited marked differences in leaf shape and size. *POTH15* OE lines also showed an increased number of branches compared to wild-type (Fig. 4C, D). Moreover, wild-type potato plants had ovate leaflets (Fig. 4F, H), whereas *POTH15* OE lines developed curved, mouse-ear-shaped leaflets (Fig 4G, I). The petiole, petiolule, and rachis of *POTH15* OE lines were also severely shortened and leaves were clustered closer to the stem (Fig. 4D, E, G, I). Thus, the leaves of *POTH15* OE lines were significantly smaller, with a bouquet of leaflets arranged on the petiole (Fig. 4G, I) in contrast to wild-type plants (Fig. 4F, H). The venation was also changed to palmate from pinnate.

**Fig. 5.**
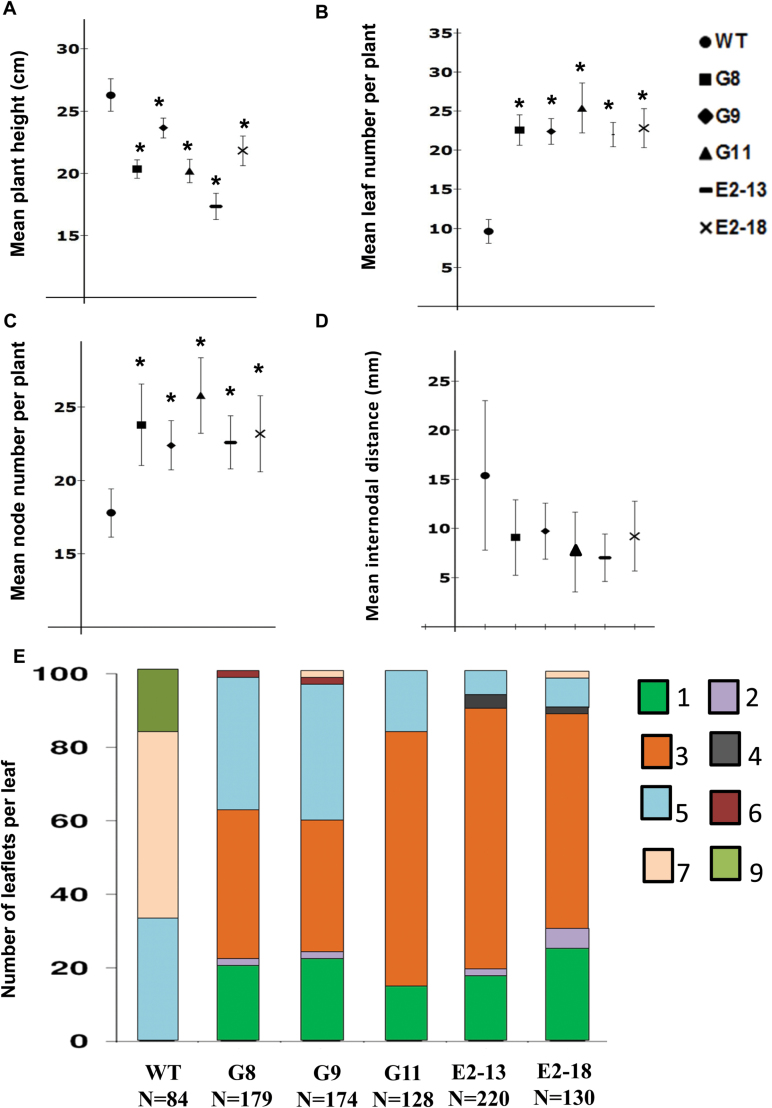
*POTH15* overexpression (OE) alters multiple morphological traits. Wild-type and *POTH15* (OE) lines (G8, G9, G11, E2-13, and E2-18) were grown for 10 weeks under LD conditions and plant height (A), number of leaves per plant (B), number of nodes per plant (C), and internodal distance (D) were measured for six individual plants per line. Data are means ± standard deviations. Leaflet number per leaf was also determined for these plants (E), where numbers 1–7 and 9 represent the average number of leaflets per leaf in wild-type and *POTH15* OE lines.

All the OE lines showed a reduction in plant height (Fig. 5A) but developed more nodes per plant compared to the wild-type (Fig. 5C). Interestingly, *POTH15* OE lines were found to retain most of the leaves on the stem in contrast to the wild-type plants, which abscised 7–8 basal leaves (Fig. 5B). The internodal distances for *POTH15* OE lines were not significantly different compared to wild-type plants; however, the variation in the internodal distance was higher in wild-type plants (Fig. 5D). Interestingly, *POTH15* OE plants had fewer leaflets per leaf than wild-type plants; the majority of the wild-type leaves had five or seven leaflets (34% or 53% of total leaves, respectively), whilst 60 to 80% of the leaves of *POTH15* OE lines had either one or three leaflets (Fig. 5E). Wild-type plants almost always had an odd number of leaflets on a given leaf whereas leaves with 2, 4, and 6 leaflets were more common in *POTH15* OE lines (Fig. 5E).

**Fig. 6.**
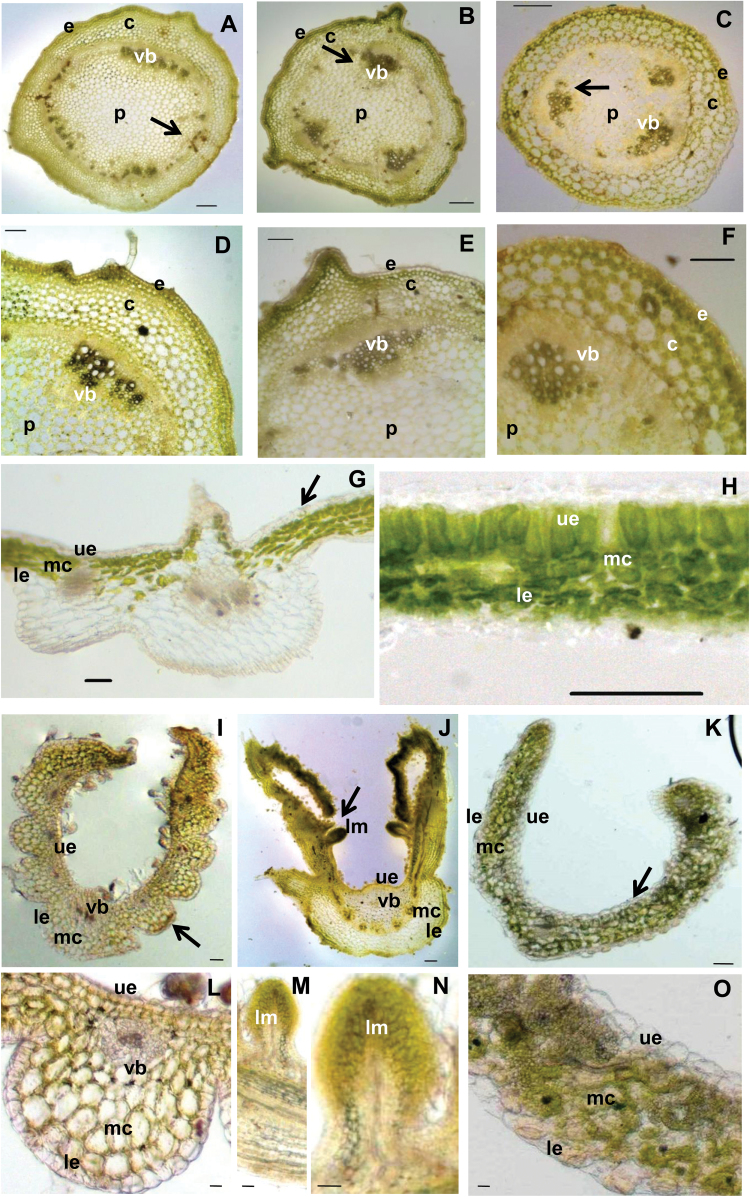
*POTH15* overexpression (OE) changes cell arrangements in stem and leaves. Cross-sections of the stems of wild-type (A, D; where D is a magnified view of A, arrow) and *POTH15* (OE) lines (B, C, E, F; where E and F are magnified views of B and C, respectively, arrows). Leaf cross-sections of wild-type (G, H; where H is a magnified view of G, arrow) and *POTH15* (OE) lines (I–O, where L–O are magnified views of I–K, respectively, arrows). Scale bars: (A–K) 100 µm; (L–O) are 20 µm. Abbreviations: e, epidermis; c, cortex region; vb, vascular tissues; p, pith region; ue, upper epidermis; mc, mesophyll cells; le, lower epidermis; lm, meristem-like structures on leaf surfaces.

Cross-sections of the stem from *POTH15* OE lines showed an alteration in cellular architecture, such as clustered vascular bundles (Fig. 6B, C, E, F) in contrast to the uniformly distributed bundles found in wild-type stems (Fig. 6A, D). Wild-type leaf cross-sections displayed well-organized, vertically packed palisade cells and parenchymatous tissue (Fig. 6G, H), whereas cellular organization in *POTH15* OE lines was found to be severely distorted, with lobing of leaflets on the abaxial side (Fig. 6I–O) along with deformation of the midvein. Remarkably, leaves of *POTH15* OE lines developed centres of meristem-like cells on the adaxial side (Fig. 6J, M, N). Similarly, *35S::POTH15* tobacco lines showed marked defects in plant development (Supplementary Figs S2–S4). *POTH15* OE lines G-8 and E2-13 and wild-type plants did not produce any tubers under LD conditions. Under SD conditions, OE line G8 produced a significant reduction in tuber yield compared to wild-type plants (Supplementary Fig. S5), whereas OE line E2-13 did not produce any tubers.

### POTH15 regulates key developmental genes

**Fig. 7.**
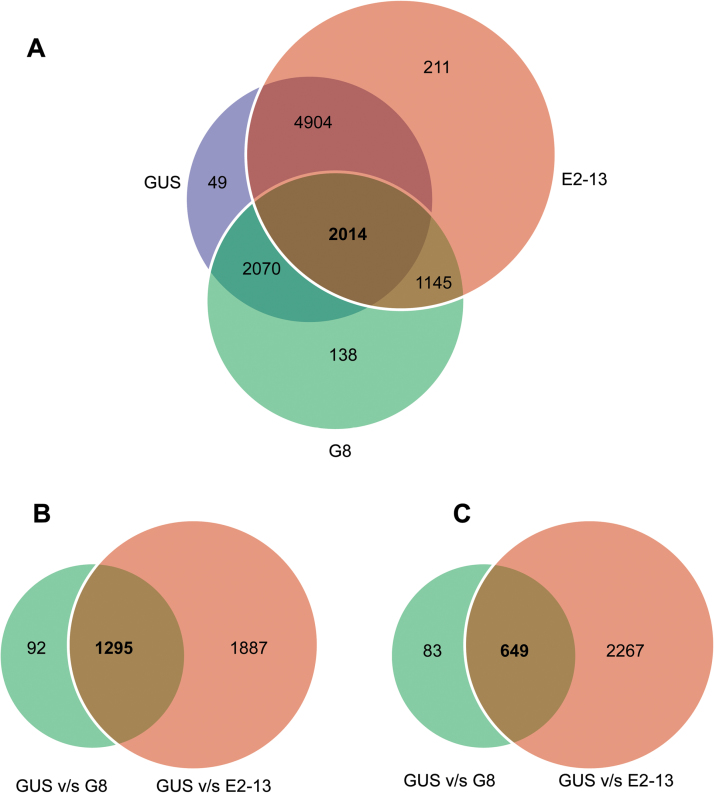
Venn diagrams showing the overlaps of numbers of differentially expressed (DE) genes in three transgenic lines identified from the RNA-seq analyses. (A) Overlaps of number of DE genes identified in three transgenic lines: *35S::GUS* (GUS), and *POTH15* OE lines G8 and E2-13 from three biological replicates. (B, C) Overlaps in up-regulated (B) and down-regulated (C) DE genes between GUS versus G8 and GUS versus E2-13 transgenic lines. Down-regulated DE genes in (C) also included 49 GUS-specific genes as shown in (A). Comparison for up- and down-regulated DE genes between G8 and E2-13 transgenic lines is shown in Supplementary Table S4.

To detect genes and pathways that are regulated by *POTH15*, we performed a transcriptome analysis using 12-week-old soil-grown long-day plants of *35S::GUS* (control) and *35S::POTH15* OE lines G8 and E2-13. Details of the number of reads obtained and the alignment rate to the potato genome for each sample are given in Supplementary Table S3. Overexpression of *POTH15* in *S. tuberosum* spp. *andigena* 7540 resulted in significant expression changes (adjusted *P*<0.05) of 8517 genes compared to *35S::GUS* control plants. Of the total differentially expressed (DE) genes, 4569 genes were up-regulated and 3550 genes were down-regulated (Fig. 7; Supplementary Table S4). Of the remaining 398 genes, 49 were specific to *35S::GUS*, 138 genes were specific G8, and 211 were specific to E2-13 (Fig. 7). Of 8517 total DE genes, our analysis revealed that 2218 genes were differentially expressed in the G8 line, whereas 5148 genes were differentially expressed in the E2-13 line (Supplementary Table S4). The difference in *POTH15* expression (Fig. 4B) and subsequent number of target genes (Supplementary Table S4) between the two *POTH15* OE lines G8 and E2-13 could be due to the varying number of gene integrations in the host plant genome or could also be the result of a position effect. Amongst the G8 and E2-13 lines, 1151 common genes were differentially expressed (Fig. 7). Further analysis suggested that there were 1387 and 683 genes significantly up- and down-regulated, respectively, in the G8 line along with 12 GUS-specific and 136 G8-specific genes. Similarly, the number of genes up- and down-regulated in the E2-13 OE line was 2654 and 2250, respectively, followed by 37 GUS-specific and 207 E2-13-specific DE genes (Supplementary Table S4). The number of DE genes common to both OE lines was found to be 2014. Further comparison between the G8 versus E2-13 OE lines showed an up-regulation of 528 genes and down-regulation of 617 genes; two genes were G8-specific whereas four genes were E2-13-specific (Fig. 7).

Gene ontology (GO) categorized unique DE genes into 24495 GO terms (Supplementary Table S7). Up-regulated genes were categorized into biological processes (4848), molecular functions (4426), and cellular components (2141). Down regulated genes were grouped into biological processes (5690), molecular functions (4598), and cellular components (2457). G8-specific genes were assigned to 54 biological processes, 47 molecular functions, and 14 cellular components in GO terms, whereas E2-13-specific genes were categorized into 77 biological processes, 89 molecular functions, and 22 cellular components. Moreover, in GUS-specific genes the number of GO terms assigned to biological processes, molecular functions, and cellular components were 15, 14, and three, respectively. Functional analysis of these genes categorized them into different metabolic processes, transport, response to abiotic and/or biotic stresses, protein metabolism, developmental processes, signaling, etc. (Table 1). The enriched categories comprise genes that are involved in hormone metabolism and hormone responses, such as ethylene responsive TFs, small auxin up-regulated RNA (SAUR) family proteins, caboxy-lyases including cytokinin riboside 5’-monophosphate phosphoribohydrolase (LOG1), LOG3, LOG4 (Supplementary Table S6) and LOG7 (Supplementary Table S5). Additionally, genes involved in signal transduction and cell cycle regulation, such as MAD2, kinesin, serine/threonine protein kinases, MAP kinases and calmodulin-binding proteins were also differentially expressed (Supplementary Table S6). Several TFs, such as homeobox proteins, NAC domain proteins, *CINCINNATA-LIKE TEOSINTE BRANCHED 1-CYCLOIDEA-PCF* (*TCP*), MADS box proteins, MYB TFs, and AP2/ERF domain-containing proteins, were also differentially expressed (Supplementary Table S6). The accession numbers for all these genes are given in Supplementary Table S10.

**Table 1.** Functional classification of genes differentially expressed between *POTH15* overexpression and wild-type plants. Genes were classified by functional categories following gene ontology (GO) terms: biological process (A), molecular functions (B), and cellular components (C). The number of GO terms assigned to each functional category are represented as a percentage, ‘X’ for upregulated and ‘Y’ for downregulated genes

A			
No.	Biological processes	X (%)	Y (%)
1	Other metabolic processes	28.61	30.95
2	Unknown biological Processes	17.49	10.32
3	Transport	11.08	4.25
4	Response to abiotic or biotic stresses	9.98	3.02
5	Protein metabolism	9.20	9.30
6	Transcription & translation	7.45	4.87
7	Other biological processes	5.38	6.70
8	Signal transduction	4.79	2.21
9	Other cellular processes	2.89	15.34
10	Developmental processes	2.50	6.68
11	DNA or RNA metabolism	0.64	6.36

B			
**No.**	**Molecular functions**	**X (%**)	**Y (%**)
1	Binding	36.42	41.87
2	Transferase	11.05	10.11
3	Oxidoreductase activity	7.00	4.78
4	Hydrolase activity	6.39	7.53
5	Kinase activity	5.94	4.35
6	Transporter	4.09	0.00
7	Catalytic activity	3.05	2.39
8	Peptidase activity	2.42	1.87
9	Ligase	1.42	1.30
10	Monooxygenase activity	1.42	0.61
11	Phosphatase activity	1.33	0.83
12	Molecular function	1.17	1.07
13	Dimerization activity	0.97	0.87
14	Lyase activity	0.95	1.63
15	Peroxidase activity	0.75	0.00
16	Dehydrogenase	0.68	0.00
17	Reductase activity	0.63	0.43
18	Isomerase activity	0.54	1.00
19	Channel activity	0.45	0.00
20	ATPase activity	0.27	0.80
21	Pectinesterase activity	0.27	0.59
22	microtubule motor activity	0.00	1.50
23	Helicase	0.00	1.07
24	Nuclease	0.00	0.76
25	Others	12.77	14.64

C			
**No.**	**Cellular components**	**X (%**)	**Y (%**)
1	Membrane	30.07	12.62
2	Nucleus	10.78	11.15
3	Chloroplast	6.54	15.22
4	Plasma membrane	6.12	4.27
5	Cytoplasm	4.86	5.98
6	Intracellular	4.34	4.48
7	Cytosol	3.97	4.60
8	Vacuole	3.32	0.77
9	Extracellular region	2.94	1.51
10	Golgi apparatus	2.80	3.54
11	ER	2.71	1.42
12	Plastid	2.43	3.05
13	Mitochondrion	2.24	3.13
14	Cell	1.91	1.14
15	Cell wall	1.77	3.38
16	Peroxisome	1.63	0.37
17	Plasmodesma	1.59	2.12
18	Apoplast	0.98	1.38
19	Ribosome	0.70	1.95
20	Thylakoid	0.70	1.02
21	Endosome	0.51	0.98
23	Exocyst	0.28	0.08
24	Phragmoplast	0.00	0.45
25	Amyloplast	0.00	0.20
26	Others	6.77	15.17

Under the molecular functions category, ~39% of up-regulated genes were categorized for enzyme activity, such as transferases, oxidoreductases, hydrolases, kinases, catalases, and peptidases. Genes with enzyme activity were also over-represented (~30%) in the list of down-regulated genes. As expected, approximately 37% of up-regulated genes and 42% of down-regulated genes had molecular function as ‘binding’, including binding to DNA, RNA, proteins, etc. (Table 1). Under the cellular component category, membrane, nucleus, chloroplast, plasma membrane, and cytoplasm were over-represented in both the list of up-regulated (~82%) and down-regulated (~57%) GO terms (Table 1). Functions for the differentially expressed genes are listed in Supplementary Table S5. To identify the pathways and developmental processes regulated by POTH15 targets, respective functions for 2014 POTH15 target genes common between two OE lines (G8 and E2-13) were retrieved from the TAIR database (https://www.arabidopsis.org) and were grouped into the following categories: hormones, cell cycle, stress (abiotic and biotic), flowering, shoot apical meristem and leaf development, growth and development, photosynthesis, photorespiration, cell wall, trichome and root development, light regulation, transcription regulation, transport, binding, and genes with unknown functions (Supplementary Table S6). Amongst 2014 common POTH15 targets, 245 genes were related to plant growth regulators, including auxin (37), ABA (65), GA (25), cytokinine (18), ethylene (49), brassinosteroid (17), salicylic acid (15), and jasmonic acid (19); 22 genes were related to flowering; 46 genes had functions in development of the shoot apical meristem and leaf; and 198 had roles in various stress responses. Other categories included cell cycle (53), growth and development (19), photosynthesis (17), photorespiration (13), cell wall (30), trichome (10), and root development (23). In addition, the genes also had roles in light regulation (33), transcription regulation (17), transport (129), and binding (114), whilst a further 663 genes were found to have unknown functions (Supplementary Table S6).

### Validation of POTH15 targets

**Fig. 8.**
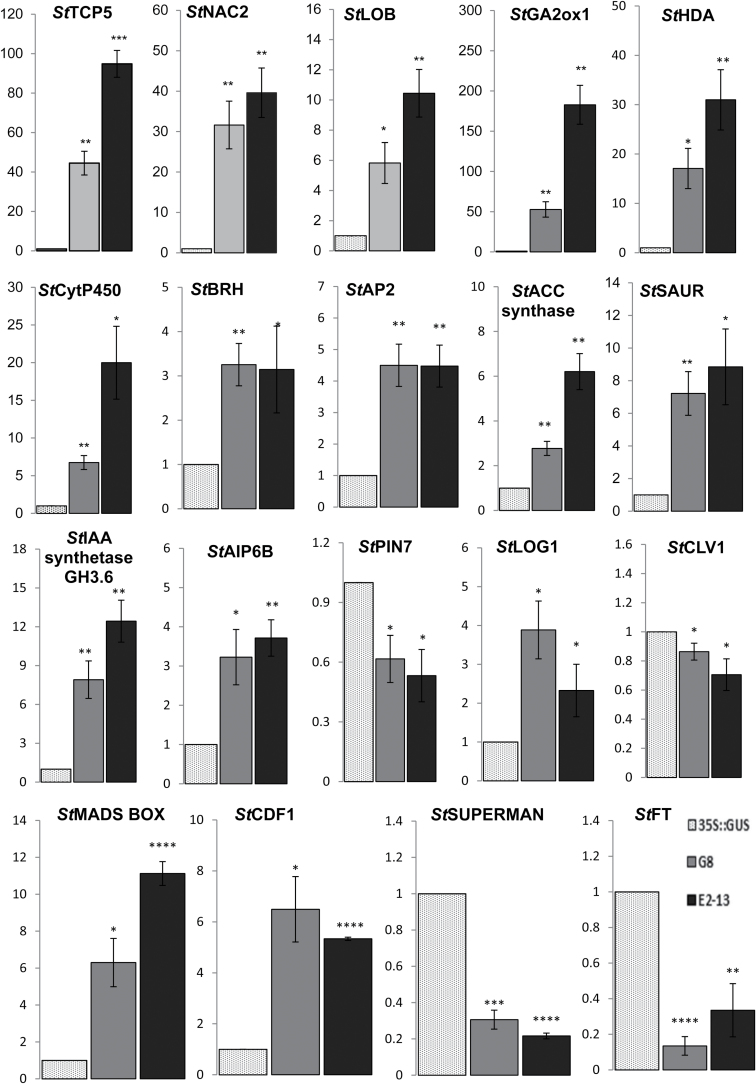
Validation of POTH15 target genes by qRT-PCR. Nineteen candidate POTH15 target genes were chosen from RNA sequencing analyses for validation. Relative mRNA levels for candidate genes in the shoot apex samples of *POTH15* overexpression lines G8 and E2-13 grown under LD conditions for 12 weeks are shown with respect to *35S::GUS* (GUS) control plants. *StActin* mRNA was used as reference for normalization. The fold-change in RNA levels was calculated as the 2^–ΔΔCt^ value relative to the mean values in the GUS sample. Error bars represent ± standard deviations from three biological replicates for each line. Data for each gene were analyzed by Student’s *t*-test separately for GUS/G8, and also for GUS/E2-13 combinations. * Represents significant difference at *P*≤0.05, ** *P*≤0.01, *** at *P*≤0.001, **** at *P*≤0.0001.

To validate the expression levels of targets from RNA-sequence data, 19 POTH15 target genes from the pool of DE genes were selected based on their known or predicted roles in plant development (Fig. 8; Supplementary Table S9). These genes were: *TCP*, *NAC domain containing protein* (*NAC2*), *LOB*, *GA2ox*, *Cytochrome P450*, *BRH* (*Brassinosteroid hydroxylase*), *SAUR*, *Histone Deacetylase* (*HDA*), auxin responsive proteins – *IAA synthetase GH3.6* and *AIP6B*, ethylene biosynthesis gene (*ACC synthase*), *CLAVATA1*, *APETALA2*, *LONELY GUY 1* (*LOG1*), *MADS BOX protein*, *CYCLING DOF FACTOR family protein* (*CDF1*), *Flowering Locus T* (*FT*), *SUPERMAN* and *PIN7* (Fig. 8; Supplementary Table S9). Out of these 19 POTH15 target genes, 15 were found to be up-regulated and the other four had down-regulation, as determined by qRT-PCR analyses (Fig. 8). The transcript levels of *StTCP5*, *StNAC2*, *StLOB*, *StGA2ox*, *StCytP450*, *StSAUR*, *StHDA*, *StIAA synthetase GH3.6*, *StAIP6B*, *StMADS BOX protein*, *StBRH*, *StLOG1*, *StCDF1*, *StAPETALA2*, and *StACC synthase* were significantly higher in both G8 and E2-13 lines as compared to 35S::GUS plants (Fig. 8). In contrast, transcript levels of *StSUPERMAN*, *StPIN7*, *StFT*, and *StCLV1* were significantly down-regulated in both OE lines compared to 35S::GUS plants (Fig. 8). The validation results for all 19 genes were consistent with the RNA sequencing data (Fig. 8; Supplementary Table S9), suggesting the data are reliable. In a random screen of 200 DE genes common between two *POTH15* overexpression lines, ~87% of them showed the presence of at least one tandem TGAC core motif in the 3.0kb promoter region (Supplementary Table S8). A number of POTH15 targets were found to have ≥ 7 tandem motifs in their 3kb promoter sequences, such as auxin-induced protein 5NG4, WRKY protein, Zeatin O-glucosyltransferase, receptor protein kinase, WRKY transcription factor, gibberellin 2-oxidase, cytochrome P450, protein AFR, class II ethylene responsive element binding factor, kinesin NACK1, zinc finger protein, ethylene-responsive transcription factor 4, transcription factor, gibberellin receptor GID1, ARF GTPase activator, tuber-specific and sucrose-responsive element binding factor, elongation factor 1-alpha, NAC domain protein, IPR003441 and SPL domain class transcription factor (Supplementary Tables S8, S10). Our search for tandem TGAC core motifs in the promoters of 15 non-DE genes showed only 6% of them to have two or more motifs, and for DE genes (200), 72% of them had two or more motifs (Supplementary Table S8). Correlation analysis showed that the degree of fold-change and the number of tandem TGAC core motifs were not correlated (*P* value =0.26; Person *r*=0.0802) (Supplementary Table S8). The average number of tandem TGAC core motifs per gene was significantly higher (*P*<0.001) in DE genes (3.01±0.16) compared to non-DE genes (0.59±0.04) (Table 2).

**Table 2.** Statistical analysis for the tandem TGAC core motif search between differentially expressed (DE) and non-DE genes. Data were analysed by Student’s *t*-test using GraphPad prism 7 (*n*=200; *P*<0.05). **** represents significant difference at *P*≤0.001

	Average number of tandem TGAC core motifs	P value	Level of significance
DE genes	3.01±0.16	<0.001	****
Non-DE genes	0.59±0.04		

## Discussion

### *POTH15* regulation

In simple-leaf species, the expression of class-I *KNOX* genes is usually confined to meristems and stems, whereas in compound-leaf species, they are expressed in leaf primordia as well (Bharathan *et al.*, 2002; Uchida *et al.*, 2007). Previously, Rosin *et al.* (2003) detected the *POTH1* mRNA in the SAM, leaf primordia, leaf lamina, developing leaflets, stolon, and stem vascular tissue in potato. Although a number of studies (e.g. Kerstetter *et al.*, 1994; Bharathan *et al.*, 2002; Uchida *et al.*, 2007) have described *KNOX* expression patterns, photoperiod-mediated regulation of *KNOX-I* transcript abundance at the tissue-specific level has not yet been reported. *Solanum tuberosum* ssp. *andigena* is photosensitive and produces tubers only under SD conditions. We examined the effect of photoperiod on the abundance of *POTH15* mRNA in a tissue-specific manner. Interestingly, we observed that *POTH15* mRNA accumulated at high levels in shoot tips and stolons under tuber-inducting SD conditions (Fig. 2). In previous studies, transcripts of *TKN2* (Kim *et al.*, 2001), *STMP* (Ham *et al.*, 2009), and *POTH1* (Mahajan *et al.*, 2012) were demonstrated to be phloem-mobile. Moreover, a truncated sequence of the *POTH15* transcript was identified in phloem of potato (Campbell *et al.*, 2008), an indication of phloem mobility. Further, *POTH15* promoter activity was detected in apical and axillary meristems, stolon tips, tuber eyes, and meristems of tuber sprouts (Fig. 3). Similar to *POTH1*, the *POTH15* promoter sequence had several light-regulatory motifs, such as GATA, GT-1, GBF5, SORLIP1, and I-boxes (Supplementary Table S2) (Terzaghi and Cashmore, 1995; Hudson and Quail, 2003; Chatterjee *et al.*, 2007). These findings suggest the potential role of *POTH15* in meristem maintenance and leaf development.

### *KNOX* overexpression phenotype

Several studies have previously demonstrated the effect of *KNOX* overexpression (OE) in diverse plant species (Muller *et al.*, 1995; Tamaoki *et al.*, 1997; Janssen *et al.*, 1998; Rosin *et al.*, 2003; Hake *et al.*, 2004; Du *et al.*, 2009; Abraham-Juarez *et al.*, 2010; Bolduc *et al.*, 2012; Tsuda *et al.*, 2014). These studies have established the role of *KNOX-I* in SAM maintenance and leaf development. Although some of the *POTH15* OE phenotypes were similar to the above mentioned studies, numerous novel phenotypes such as a complete reduction of the petiole, petiolule, and rachis of the leaf, bouquet of leaflets on the petiole (Fig. 4D, E, G, I), increased number of branches (Fig. 4D), and clustered vascular bundles (Fig. 6B, C) in the OE lines were also observed compared to wild-type plants (Figs 4C, F, H and 6A). Occasionally, we also observed development of ectopic leaf meristems on the adaxial side of the older leaves (Fig. 6J, M, N). Ectopic expression of *KNOX-I* genes has previously been shown to either enhance or block leaflet formation depending on the developmental stage of the leaf and the competency of the cells to respond to leaflet-promoting signals (Shani *et al.*, 2009; Hay and Tsiantis, 2010). For example, when *Kn1* was overexpressed in tomato, the normally dissected leaves having 8 to 16 leaflets became severely dissected with up to 1000 leaflets (Hareven *et al.*, 1996; Janssen *et al.*, 1998). In contrast to this, we observed that *POTH15* OE lines in potato showed a decrease in the number of leaflets per leaf (Fig. 5E), but the leaflets were lobed (Fig. 4G, I) and they produced multiple secondary leaflets. Thus, overexpression of *POTH15* in potato and tobacco altered multiple morphological traits.

### POTH15 targets

As KNOX proteins act as transcription factors, identifying their target genes is imperative to understand how *KNOX* genes can regulate diverse developmental processes in plants. In previous studies, *KNOX-I* TFs have been shown to regulate the levels of GA and cytokinin (Sakamoto *et al.*, 2001; Hay *et al.*, 2002; Chen *et al.*, 2004; Yanai *et al.*, 2005; Bolduc and Hake, 2009) and the biosynthesis of lignin (Mele *et al.*, 2003; Hay and Tsiantis, 2010). A screen for *STM* targets revealed *CUC1* (*CUP SHAPED COTYLEDON 1*) is a direct target of *STM* in Arabidopsis (Spinelli *et al.*, 2011). Another interesting study by Bolduc *et al.* (2012) demonstrated that direct targets of *Kn1* include other homeobox and hormone metabolism genes. Recently, Tsuda *et al.* (2014) also showed that a rice *KNOX-I* gene *OSH1* targets brassinosteroid catabolism genes and regulates SAM functions. In spite of their significance in plant development, no screen for *KNOX* target genes in potato has been reported yet. To avoid bias, two different transgenic lines having high (E2-13) and moderate (G8) levels of *POTH15* expression (Fig. 4B) were subjected to RNA sequencing as well as for the validation of selected POTH15 target genes. Our comparative transcriptome analysis of *35S::GUS* and two *POTH15* OE potato lines identified >6000 differentially expressed genes. Functional analysis of these genes revealed their involvement in key biological processes such as cellular, metabolic processes, response to hormones and biotic/abiotic stresses, transcription regulation, transport, signal transduction pathways and many others (Table 1), suggesting that *POTH15* functions in diverse developmental processes in potato. As OE lines of *POTH15* were used for RNA sequencing analysis, the list of differentially expressed genes could include both direct as well as indirect targets of POTH15.

RNA sequencing analyses have identified many interesting targets of POTH15 potentially involved in SAM and leaf development, flowering, plant defence, and hormone metabolism (Fig. 8; Supplementary Tables S5, 6). Important leaf development-related genes such as *St*TCP5 (Li et al., 2012) and *St*LOB (Timmermans *et al.*, 1999) were also found to be differentially expressed in *POTH15* OE lines. Flowering-related genes such as *St*NAC2 (Aida et al., 1997, 1999), *St*SUPERMAN (Nandi *et al.*, 2000) *St*Apetala2, (Okamuro *et al.*, 1997), *St*MADS BOX protein (Steffen *et al.*, 2007) and Flowering Locus T (*St*FT) (Navarro *et al.*, 2011) were also found to be POTH15 targets (Fig. 8). Moreover, genes involved in hormonal metabolism and signaling [e.g. auxin-responsive and transport-related genes such as *St*SAUR, *St*AIP6B, *St*IAA synthetase, and *St*PIN7 (Woodward and Bartel, 2005; Jain and Khurana, 2009; Kant *et al.*, 2009; Wu *et al.*, 2012; Pattison and Catelá, 2012); cytokinine biosynthesis gene *St*LOG1 (Kurakawa *et al.*, 2007); GA catabolism gene *St*GA2ox (Kloosterman *et al.*, 2007; Bolduc and Hake, 2009); ethylene biosynthesis gene *St*ACC synthase (Destéfano-Beltrán *et al.*, 1995); and brassinosteroid metabolism-related genes including *St*CytP450 and *St*BRH (brassinosteroid hydroxylase)] were enriched in the list of POTH15 targets (Fig. 8). Some of these targets have previously been shown as *KNOX-I* targets by Bolduc *et al.* (2012) and Tsuda *et al.* (2014). Among the 2014 common targets of POTH15 that we analyzed for hormone metabolism, genes having functions in ABA metabolism were the most prominent (65), followed by ethylene (49), auxin (37), GA (25), and cytokinine (18) (Supplementary Table S6). There were also genes that had functions in the metabolism of jasmonic acid (19), brassinosteroid (17), and salicylic acid (15) (Supplementary Table S6). Moreover, we could find 22 genes related to flowering, 46 genes had functions in development of the shoot apical meristem and leaf, and 198 had roles in various stress responses. In addition, POTH15 targets also had functions in various other processes such as the cell cycle (53), growth and development (19), photosynthesis (17), photorespiration (13), development of cell walls (30), trichomes (10) and roots (23), light regulation (33), transcription regulation (17), transport (129), and binding (114) (Supplementary Table S6). Thus, it appears that *POTH15* regulates diverse developmental processes in potato. Recently, Sharma *et al.* (2016) have shown that the abundant target genes of *St*BEL5 were associated with metabolism of auxin, ABA and ethylene, flowering, growth and development, transcription regulation, and signal transduction. These findings along with our observations further suggest that *St*BEL5 and POTH15 may share numerous common targets genes.

In qRT-PCR validation analyses, RNA harvested from shoot apex samples of *POTH15* OE lines showed a significant increase in *StGA2ox1* transcript levels (>50-fold) compared to *35S::GUS* plants (Fig. 8). This is consistent with the work of Bolduc and Hake (2009) in maize where it was shown that *Kn1* (a class-I KNOX) up-regulates *GA2ox1* expression in the SAM to maintain a boundary between meristem cell identity and rapidly elongating cells. *KNOX* genes are known to positively regulate the expression of NAC-domain transcription factors such as CUC 1–3, which are involved in organ-boundary maintenance (Aida et al., 1997, 1999; Vroemen *et al.*, 2003; Hibara *et al.*, 2006). Transcript abundance of NAC-domain transcription factor *StNAC2* was significantly increased (>30-fold) in both the *POTH15* OE lines (Fig. 8), which is similar to previous reports (Takada *et al.*, 2001; Hake *et al.*, 2004; Blein *et al.*, 2008; Hay and Tsiantis, 2010). SAUR, IAA synthetase GH3.6 and AIP6B are anticipated to be involved in auxin signaling and stress defence responses (Woodward and Bartel, 2005; Jain and Khurana, 2009; Kant *et al.*, 2009; Wu *et al.*, 2012). Our results show that *POTH15* OE lines had an increase in *StSAUR* (>6-fold), *StAIP6B* (>3-fold), and *StIAA synthetase GH3.6* (>7-fold) transcript levels, whereas the level of auxin efflux facilitator (*StPIN7*) transcript was down-regulated (Fig. 8), suggesting a possible role of *POTH15* in auxin signaling as well as in auxin transport pathways. A previous study demonstrated that class-I *KNOX* TFs up-regulate *CytP450* genes associated in BR catabolism, and are involved in cell elongation and cell wall modification (Donaldson and Luster, 1991; Sun *et al.*, 2010; Tsuda *et al.*, 2014). Similar to the observation of Tsuda *et al.* (2014), *StCytP450* and *Brassinosteroid hydroxylase* (*StBRH*) transcript levels were significantly higher in *POTH15* OE lines compared to *35S::GUS* plants (Fig. 8), indicating that *POTH15* regulates BR metabolism by regulating the expression of *CytP450* genes. Similarly, Sharma *et al.* (2016) have shown that genes involved in BR metabolism were also enriched in the list of *St*BEL5 targets, implicating a possible KNOX–BEL interaction. Chen *et al.* (2004) have demonstrated that the KNOX (POTH1) – BEL (*St*BEL5) heterodimer binds to a tandem TTGAC motif in the promoter of target gene in order to regulate developmental processes in potato. Approximately 92% of 200 *St*BEL5 targets contained at least one tandem TGAC core motif (Sharma *et al.*, 2016). In an effort to find if tandem TGAC core motifs are also present in *KNOX* target genes, our search revealed that 173 out of 200 target genes have a characteristic TGAC core motif within 3.0kb in the upstream promoter sequence (Supplementary Table S8) suggesting a possible relationship between the KNOX–BEL heterodimer and their targets. Moreover, it was also evident from the data in Table 2 that DE genes had a significantly higher number of tandem TGAC core motifs compared to non-DE genes, suggesting that POTH15 may interact with its targets through this motif and regulate gene expression. However, correlation analysis showed that the degree of fold-change and the number of tandem TGAC core motifs were not related. For example, the presence of even one tandem TGAC core motif in the promoter of a target genes is sometimes enough to cause a greater change in the respective target gene expression and vice versa (Supplementary Table S8). To obtain further insights into the role of *POTH15* and to identify direct targets would be part of our future investigations. In summary, this study demonstrates that *POTH15* regulates a wide range of target genes involved in diverse functions, and provides new knowledge on the role of *POTH15* in regulating potato plant development.

## Supplementary data

Supplementary data are available at *JXB* online.

Figure S1. *KNOX* genes in potato.

Figure S2. *POTH15* overexpression dramatically changes the plant architecture in tobacco.

Figure S3. *POTH15* overexpression in tabacco changes cell arrangements in the stem.

Figure S4. *POTH15* overexpression in tobacco changes cell arrangements in the leaves.

Figure S5. *POTH15* overexpression reduces overall tuber yield in potato under SD conditions.

Table S1. List of primers.

Table S2. List of regulatory motifs in the *POTH15* promoter predicted by PlantPan.

Table S3. Number of reads obtained in the RNA-sequencing analysis and alignment rate to the potato genome.

Table S4. Summary of differentially expressed genes obtained from RNA-sequencing analysis.

Table S5. List of differentially expressed genes in *POTH15* OE lines G8 and E2-13 with respect to *35S::GUS* line controls, as shown from RNA-sequencing analysis.

Table S6. List of DE genes common between G8 and E2-13 *POTH15* OE lines.

Table S7. Gene ontology annotations for DE genes.

Table S8. Tandem TGAC core motif search for 200 random *POTH15* targets common between G8 and E2-13 *POTH15* OE lines.

Table S9. RNA-sequencing results for validated *POTH15* target genes.

Table S10. List of genes with their accession numbers.

Supplementary Data
