# Supplementary material for: Regulation, overexpression, and target gene identification of Potato Homeobox 15 (POTH15) – a class-I KNOX gene in potato
Source: J Exp Bot. 2016 May 23;67(14):4255–72. doi: 10.1093/jxb/erw205 (PMC5301930; doi:10.1093/jxb/erw205)
Supplement: Supplementary Data [file supp_67_14_4255__index.html]

Regulation, overexpression, and target gene identification of Potato Homeobox 15 (POTH15) – a class-I KNOX gene in potato — Regulation, overexpression, and target gene identification of Potato Homeobox 15 (POTH15) – a class-I KNOX gene in potato — Supplementary Data 

# Regulation, overexpression, and target gene identification of *Potato Homeobox 15* (*POTH15*) – a class-I *KNOX* gene in potato

## Supplementary Data

Data files

- Supplementary\_table\_S1.xlsx - Supplementary Data
- supplementary\_tables\_S2\_S4\_figures\_S1\_S5.pdf - Supplementary Data
- supplementary\_tables\_S5.xlsx - Supplementary Data
- supplementary\_tables\_S6.xlsx - Supplementary Data
- supplementary\_tables\_S7.xls - Supplementary Data
- supplementary\_tables\_S8.xlsx - Supplementary Data
- supplementary\_tables\_S9.xlsx - Supplementary Data
- supplementary\_tables\_S10.xlsx - Supplementary Data
