# Supplementary material for: Regulation, overexpression, and target gene identification of Potato Homeobox 15 (POTH15) – a class-I KNOX gene in potato
Source: J Exp Bot. 2016 May 23;67(14):4255–72. doi: 10.1093/jxb/erw205 (PMC5301930; doi:10.1093/jxb/erw205)
Supplement: Supplementary Data [file supp_erw205_supplementary_tables_S2_S4_figures_S1_S5.pdf]

Regulation, over-expression and target gene identification of *Potato Homeobox 15* (*POTH15*) - a class-I *KNOX* gene in potato

Ameya S. Mahajan[1], Kirtikumar R. Kondhare[1], Mohit P. Rajabhoj[2], Amit Kumar[1], Tejashree Ghate[3], Nevedha Ravindran[4], Farhat Habib[1], Sundaresha Siddappa[5] and Anjan K. Banerjee[1]*

Table S2. List of regulatory motifs in *POTH15* promoter predicted by PlantPan (Chang *et al*., 2008).

**Motif**	**Number**
AGL-3	2
AG	10
Athb-1	30
ANT	1
Athb-5	28
Athb-9	6
CDC-5	2
RAV1	12
ACGTATERD1	2
ANAERO1CONSENSUS	2
AP1	4
ARR10	7
ARR1AT	34
AtMYC2	1
Bellringer	7
AGL-15	20
CCA1	2
ATHB33	74
DPBF-1	1
GARE	2
GATABOX	23
GBF5	3
GT1CONSENSUS	45
IBOX	2
L1BOX	1
MYB1	13
MYCATERD1	1
MYCATRD22	1
MYCCONSENSUSAT	8
POLASIG1	12
PREATPRODH	3
SORLIP1	1
SRE	3
SURE	2
SURECOREATSULTR11	3
TAAAGSTKST1	11
TBOX	1
WBOX	2

Table S3. Number of reads obtained in the RNA-seq analysis and aligned to the potato genome.

**Transgenic line**	**Sample**	**Total no. of reads obtained**	**Mean read length (bp)**	**Overall alignment rate**
Control line (35S::GUS)	GUS-1A	35,326,000	129	46.6%
	GUS-2A	20,095,643	131	46.9%
POTH15 OE line (G8)	G8-1A	32,012,002	138	33.9%
	G8-2A	37,166,422	143	33.9%
	G8-3A	53,506,123	127	51.5%
POTH15 OE line (E2-13)	E2-13-1A	19,644,473	131	48.9%
	E2-13-2A	35,906,847	116	47.4%
	E2-13-3A	48,460,358	128	45.7%

Table S4. Summary of differentially expressed (DE) genes obtained from RNA-seq analysis.

**Comparison**	**Category**	**No. of DE genes**
GUS vs G8	Up-regulated	1387
	Down-regulated	683
	GUS specific	12
	G8 specific	136
	Total	2218
GUS vs E2-13	Up-regulated	2654
	Down-regulated	2250
	GUS specific	37
	E2-13 specific	207
	Total	5148
G8 vs E2-13	Up-regulated	528
	Down-regulated	617
	G8 specific	2
	E2-13 specific	4
	Total	1151
	Common between GUS vs G8 and GUS vs E2-13	2014
	All up-regulated	4569
	All down-regulated	3550
	Either GUS or G8 or E2-13 specific	398
	**Total DE genes**	**8517**

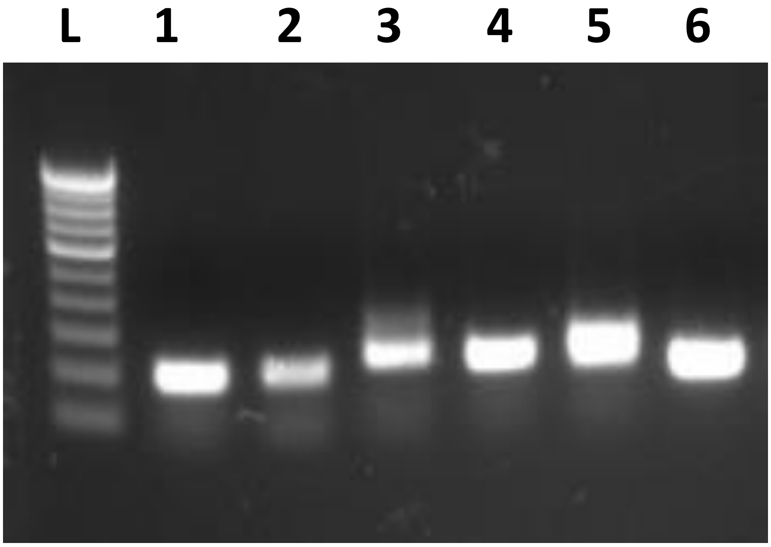

L – Step up 100bp ladder

1 – POTH20 (Potato Homeobox 20) (KP335125 and KP335126)

2 – POTH15 (Potato Homeobox 15) (KJ477687)

3 – *St*Petroselinum (KJ477688)

4 - *St*Kn1 (KJ477691)

5 – *St*Hox1 (KJ477689)

6 – *St*Hox2 (KJ477690)

Fig. S1. KNOX genes in potato. The putative sequences of KNOX genes in potato were obtained from PGSC database. The expressions of these genes were validated by RT-PCR. Further, the full-length transcript sequence for each of the genes was obtained by 5'-RACE and the sequences were validated and deposited in NCBI. The accession numbers are mentioned in brackets.

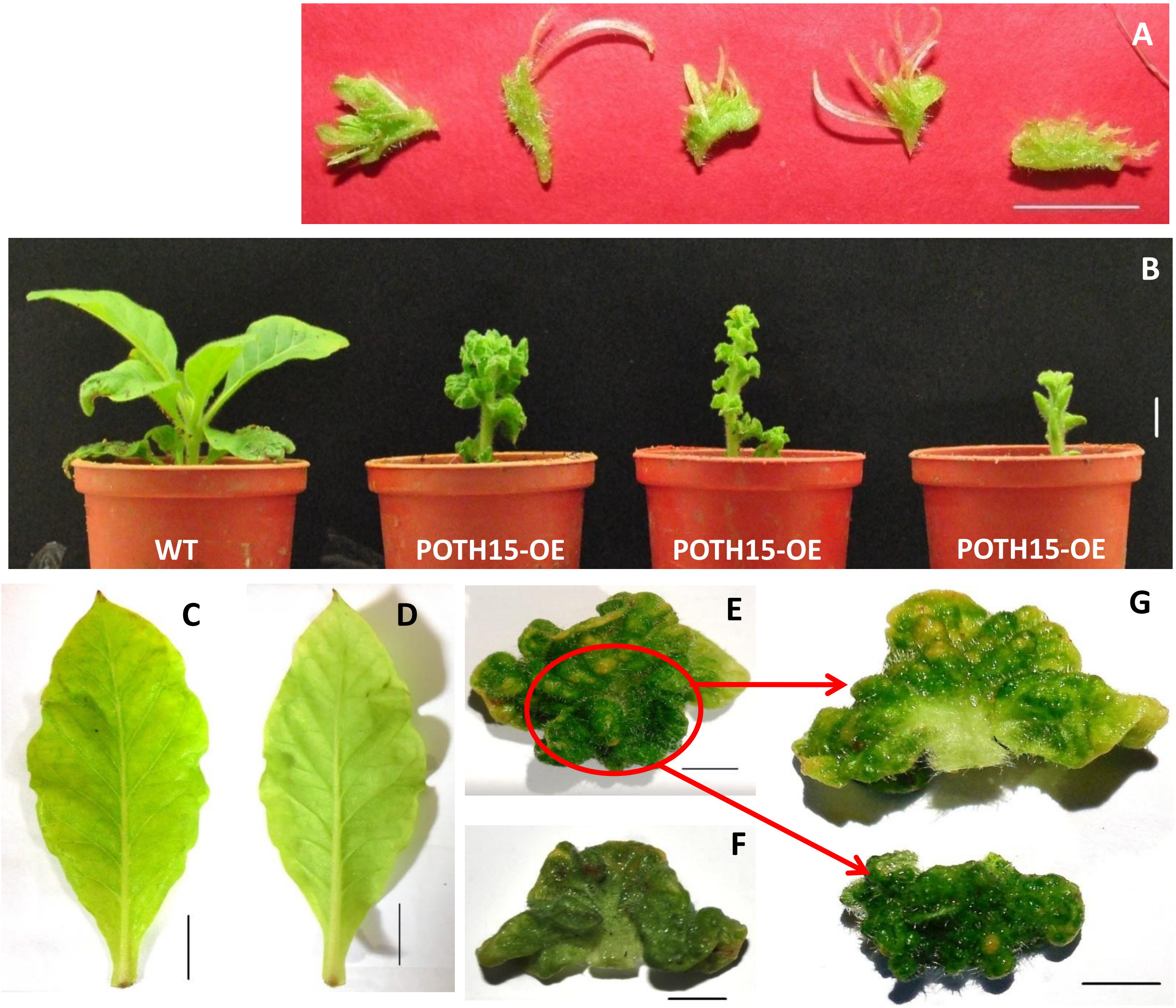

Fig. S2. POTH15 over-expression drastically changes the plant architecture in tobacco (*Nicotiana tabacum* var *Petite Havana*). Leaves of *in vitro* grown POTH15 over-expression lines of tobacco (A). Eight weeks old plants of wild-type (WT) and POTH15 over-expression (B) tobacco lines. The dorsal and ventral view of the leaves from wild-type (C and D) and POTH15 over-expression lines (E to G where G is a magnified view of E with two fused leaves separated). The mature leaves of POTH15 over-expression lines of tobacco showed leaf-on-leaf phenotype. The scale bars in A, B, C and D are 1 cm and scale bars in E, F and G are 5 mm.

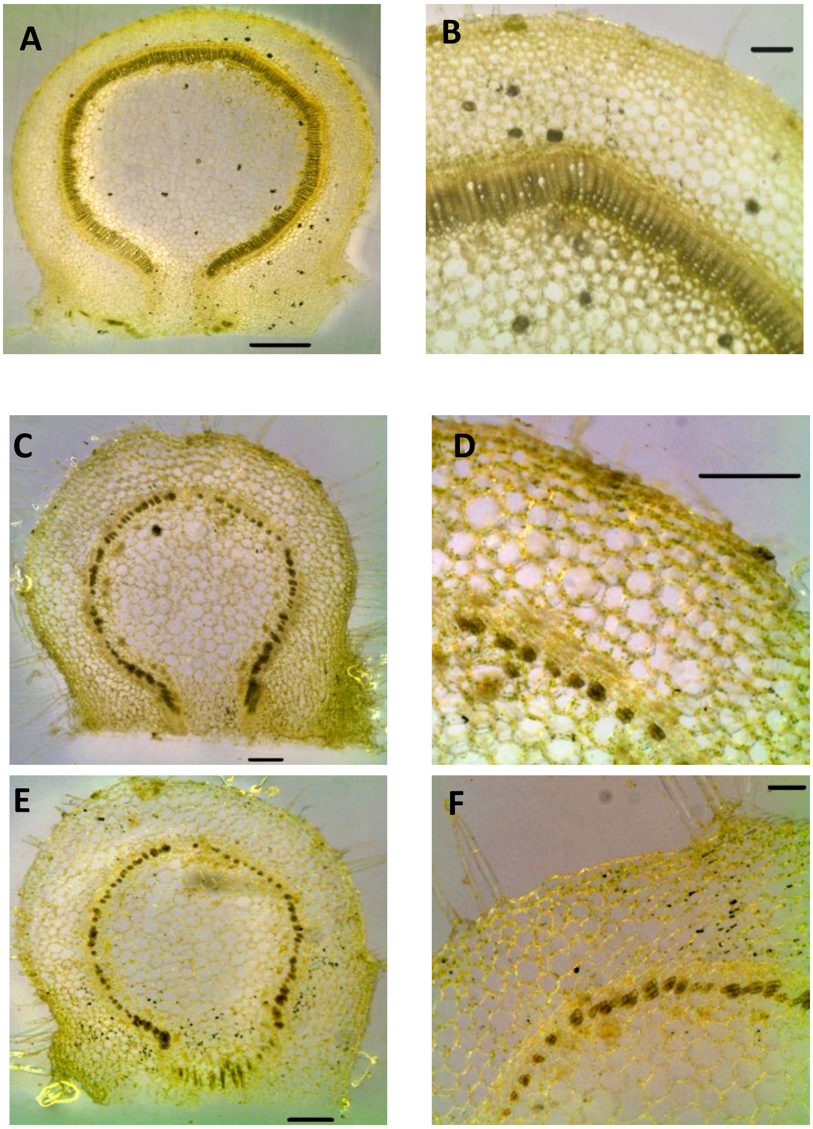

Fig. S3. POTH15 over-expression in tobacco changes cell arrangements in stem. Cross sections of stem of the wild-type (A) and POTH15 over-expression (C and E) lines of tobacco. B, D and F are magnified views of A, C and E, respectively. Scale bars in A, C, D and E are 50 microns, in B and F are 10 microns.

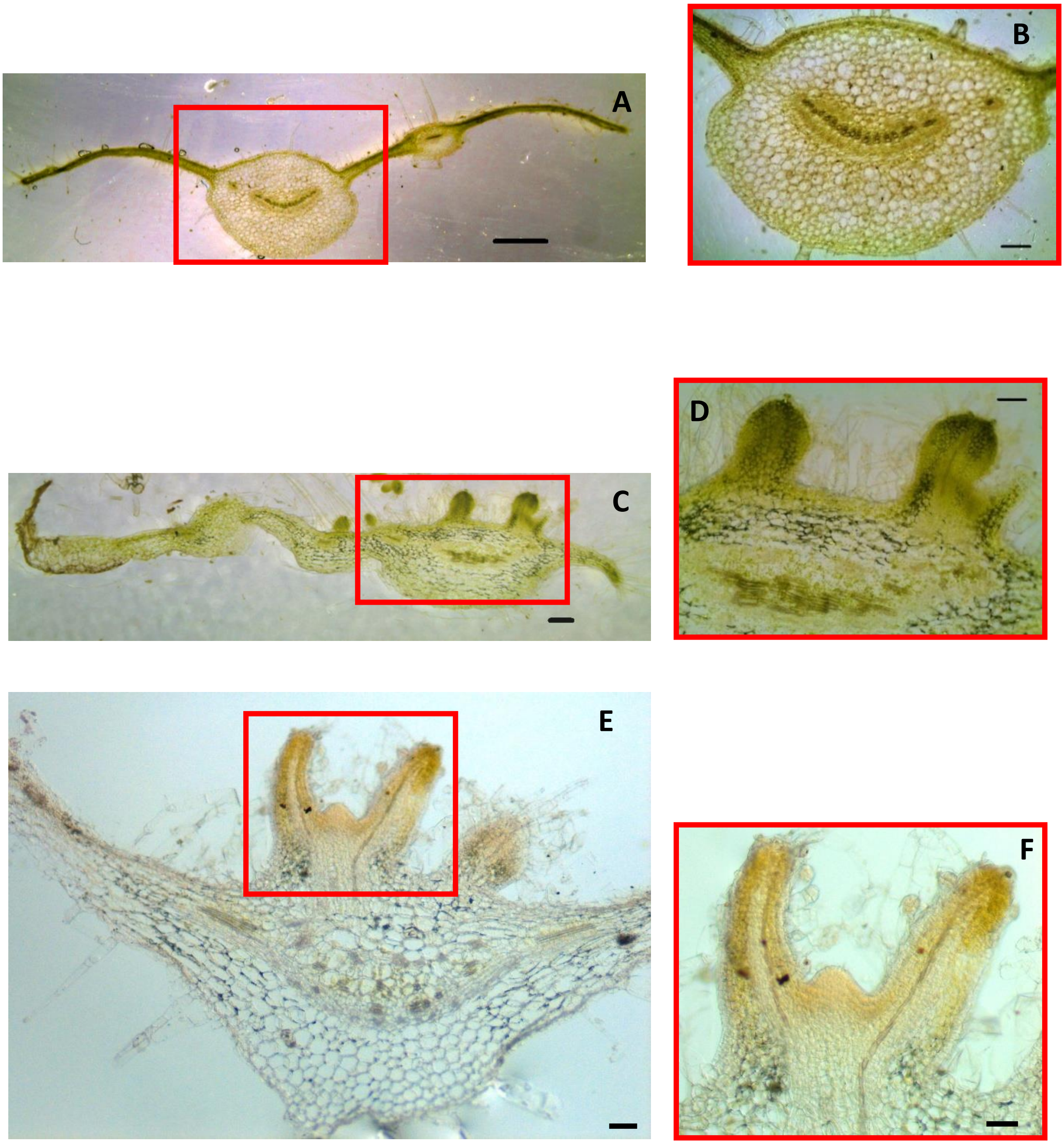

Fig. S4. POTH15 over-expression in tobacco changes cell arrangements in leaves. Cross sections of the leaves of the wild type (A) and POTH15 over-expression (C and E) lines of tobacco. B, D and F are magnified views of A, C and E, respectively. Meristem-like structures (D and F) were observed on POTH15 over-expression leaves.

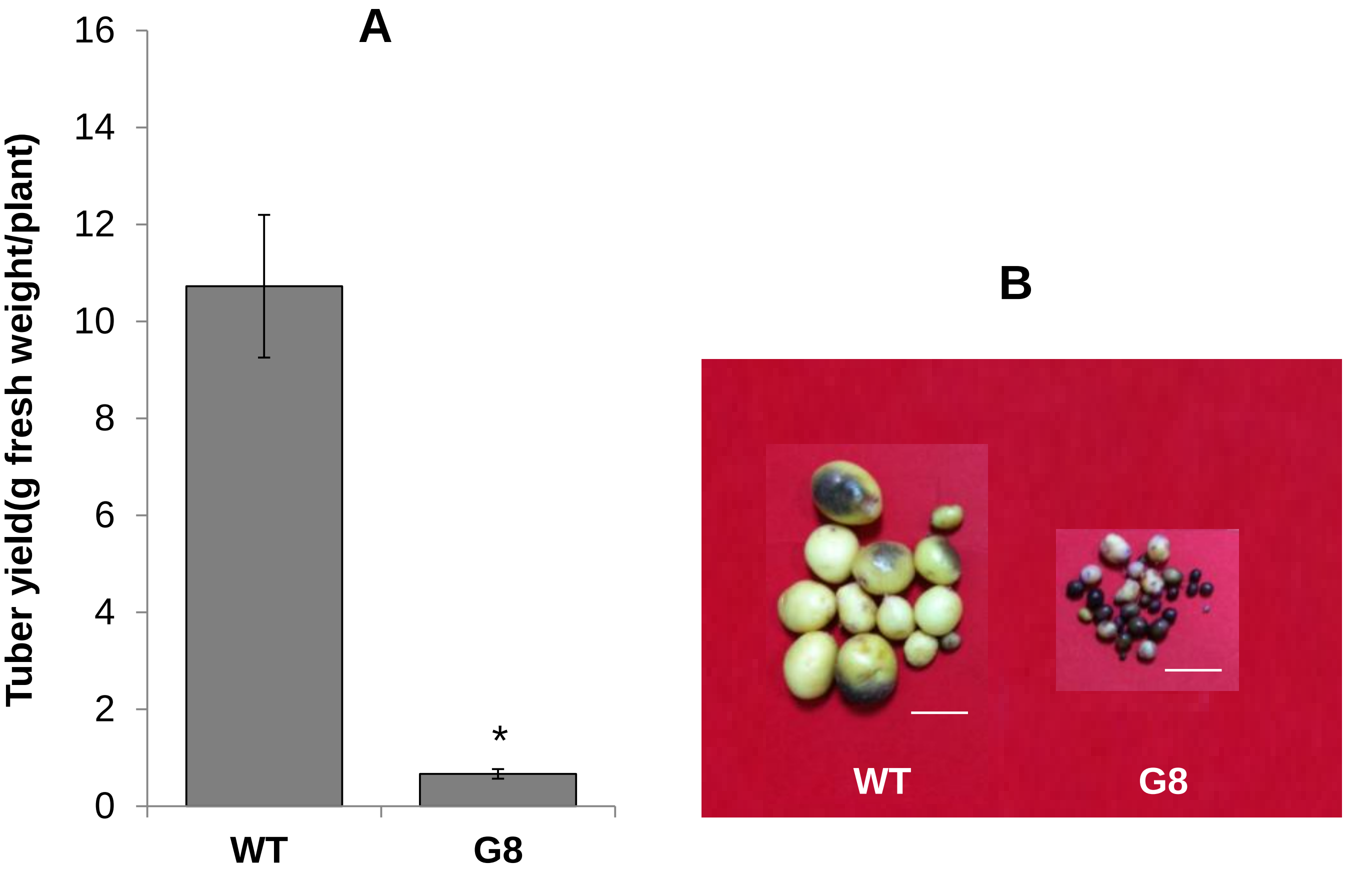

Fig. S5. POTH15 over-expression reduces overall tuber yield under SD conditions. Wild-type plants (WT) and POTH15 over-expression lines (G8 and E2-13) were grown in soil under LD conditions (16 h light, 8 h dark with light intensity of 300 mmol m^{-2} s^{-1} at 22±1 °C) for 8 weeks, followed by 4 weeks under SD or LD conditions. POTH15 over-expression line- G8 showed reduced tuber yield compared to wild-type (WT) (B). The tubers depicted in (C) are the pooled harvest of six plants per line. POTH15 over-expression lines (G8 and E2-13) as well as WT did not produce tubers under LD conditions. Under SD conditions, over-expression line E2-13 did not produce tubers. Data were analysed by one way ANOVA. Error bars represent (±) standard deviations for six biological replicates. Asterisk * indicates significant difference at $P \leq 0.05$.Scale bars in C are 1 cm.
